# Work of the Heaviest Week of the War

**Published:** 1914-11-07

**Authors:** 


### THE INFLUX OF WOUNDED AT LEICESTER.

### Work of the Heaviest Week of the War.

REFERENCE was made in last week's report to the fact that the patients at Leicester Royal Infirmary were diminishing as the result of transfers to the hospitals at Wolverhampton, Loughborough, Melton Mowbray, and other towns. This week the accommodation of the base hospital has been taxed to its full capacity by the arrival of two batches of Belgian soldiers direct from West Flanders. The first convoy of 200 patients arrived early on Wednesday morning, October 28, in two ambulance trains from Southampton, about half of them being "stretcher" or "cot" cases. Awaiting the arrival of the trains at Leicester on a grey cold early morning were the County Director Voluntary Aid Detachments, Mr. A. W. Faire, and his assistant, Mr. W. F. Seymour, Lieutenant-Colonel L. K. Harrison, commandant of the base hospital, and members of his staff, Lieutenant-Colonel W. Pemberton Peake, in charge of the ambulance transport, and members of the Royal Red Cross Society and St. John Ambulance staffs. As on former occasions, the motor conveyances were marshalled by Mr. Reginald Corah, and removed the patients from the train to the base hospital.

Scarcely had this been effected when news was received from Southampton that a further ambulance train with seventeen Belgian officers and 161 men had been dispatched. To a less able administrator than Lieutenant-Colonel Harrison the intimation would have been a source of much anxiety, owing to the fact that there were few more than 100 beds at the base hospital for their accommodation, and no time was available for the transfer of semi-convalescent patients to other hospitals. He, however, at once sought the co-operation of the Leicester Royal Infirmary, with the result that at 7.30 A.M. the resources of that institution were put into operation for the reception of seventy patients. Only one ward of that institution was available at the time, but necessity providing the means, no time was lost by Miss Vincent, the matron, and the staff of the institution in getting the only available building, the out-patients' department, fitted up for the reception of patients. Instructions were at once given for the customary daily work of the department, which commences at 8.30, to be transferred to the casualty department; all the available beds and mattresses were collected, the floors thoroughly washed down, supplies obtained, and by 10 o'clock the transformation of the department had been performed. The willing co-operation of Dr. Bond, the members of the resident medical staff, and of several practitioners in the town was a feature of the preparations. But disappointment was in store, for at 10.20 came the news from Lieutenant-Colonel Harrison that the train had gone to another centre. The tension on Miss Vincent, the nurses, and the staff of the institution had been acute, but one and all felt that the effort had been of the utmost value from a disciplinary point of view.

At 5.30 the following evening news came through that an ambulance train had left Southampton at 4.10 with 150 wounded Belgian soldiers, whose arrival was to be expected at 9 o'clock. Half an hour afterwards the train arrived, sixty cases being sent to the Leicester Royal Infirmary and the remainder to the base hospital, the full number of available beds in that institution being thus occupied. Major Blakesley, Major Crosby, and Captain Cumberlidge, all members of the staff of the infirmary, were charged with the initial medical care of the patients, who, one by one, with the co-operation of Mr. Bond and the resident medical staff, and the assistance of Dr. Holyoak and Dr. Everard Harrison, practitioners, who kindly volunteered their services, were examined, dressed, and treated by midnight. Early the following morning visits were paid by the staff surgeons referred to for further medical examinations, when it was unfortunately found that three severe cases of shrapnel wounds needed operative treatment of a major character in the well-appointed and self-contained building which formed the temporary hospital.

During the whole period which passed between the first indication of the need for the infirmary's co-operation until the last patient had been received, the advice and counsel of Mr. Bond were invaluable, as was the kindness of the Mayoress, Mrs. Russell Frears, in providing a sufficient supply of flannel garments. The services of a number of Belgian residents living temporarily in the town in acting as interpreters were most useful. Major Cecil Marriott and Major Sloane, both members of the honorary staff of the infirmary, have been mobilised for the treatment of the patients, and the staff of the base hospital has been further increased by the mobilisation of Major Horron Davies and Captains Slight and Waite. Sister Mills (out-patient sister) has been placed in charge of the temporary hospital at the infirmary. Lieut.-Col. Peake kindly detached three male orderlies from his staff (which is shortly to be dispatched to the seat of war) to assist in the work at the infirmary.

